# Stigma and the Inverse *Care* Law: Experiences of ‘Care’ for People Living in Marginalised Conditions

**DOI:** 10.1111/1467-9566.70000

**Published:** 2025-01-19

**Authors:** Michelle Addison, Steph Scott, Clare Bambra, Monique Lhussier

**Affiliations:** ^1^ Department of Sociology Durham University Durham UK; ^2^ Faculty of Medical Sciences Population Health Sciences Institute Newcastle University Newcastle upon Tyne UK; ^3^ Department of Social Work, Education and Community Wellbeing Northumbria University Newcastle upon Tyne UK

**Keywords:** health inequalities, healthcare, illicit drugs, inverse care law, marginalisation, stigma

## Abstract

This paper explores the connection between stigma and the Inverse Care Law (ICL) by focussing on the idea that people who have the greatest needs often have the least support from healthcare services. Twenty‐four semi‐structured interviews were undertaken with people who used class A & B illicit drugs, in the northeast of England. Many of the people in this study who used illicit drugs were not able to access quality healthcare in a timely way to meet their needs because of structural and relational stigma. We discuss four themes: (i) pressure on health services and long waiting lists, (ii) sensitivity to compassion fatigue from staff and impacts on engagement, (iii) complex systems that are difficult to navigate and (iv) stigma and drug use. These themes illuminate the harms of stigma and support Tudor Hart's ICL. Stigma is a key contributor to the inverse experience of good quality healthcare and requires greater attention from policymakers and practitioners. The structural and relational aspects of stigma embedded in healthcare are central to the ICL and reproduce inequities in *access to* and *experience of* good quality healthcare, which in turn impacts health inequalities.

## 1 Introduction

Life expectancy for people living in deprived communities in England has begun to reverse for the first time in over a century (Marmot et al. 2020; NHS England 2023b; The King's Fund 2024b). Populations that are marginalised and minoritised are now spending more of their life in ill‐health (NHS England 2023b; Marmot et al. 2020). This is unfair and preventable. The multiple causes of this deterioration in health are complex, but scholars recognise that availability of good quality healthcare plays a fundamental role in how long a person will live, and how many years that person will be able to spend in good health (Braithwaite et al. 2017; Darzi 2024; Dubbin, Chang, and Shim 2013). Despite this, there is strong evidence that highlights systemic inequities in access to health and care services (NHS England 2023b), which can have disastrous consequences for people living in deprived areas and experiencing poor health (Dixon‐Woods et al. 2006). To unpack this critical problem further, we explore the connection between stigma and Tudor Hart's Inverse Care Law (ICL) (Tudor Hart 1971) to address the question: why are groups who are marginalised still underserved by good quality healthcare provision in the UK? In doing so, our aim is to explore the role of stigma in perpetuating health inequities in support from, and experiences within, health and care services for marginalised groups. To achieve this aim, we focus on people who use (class A & B illicit) drugs and their experiences of marginalisation and stigma within and across health and social care.

Populations that are marginalised are chronically underserved by good quality healthcare that can fully meet their needs (Lowther‐Payne et al. 2023; Tudor Hart 1971). This persistent problem constitutes the Inverse Care Law (ICL) which, as outlined by Tudor Hart, states that:

The availability of good medical care tends to vary inversely with the need for it in the population served. This inverse care law operates more completely where medical care is most exposed to market forces, and less so where such exposure is reduced.

(Tudor Hart 1971, 405)

Although the ICL was proposed over 50 years ago, it is still regarded as very current and has resonance today (Darzi 2024). There has been much evidence gathered which supports Tudor Hart's argument (Cooper 2010; Darzi 2024; Goodair and Reeves 2022; Marmot 2018; The Lancet 2021). For instance, *The Health Foundation* conducted analysis of policies aimed at improving general practice since 1990 and found that, even after accounting for differences in health care needs, ‘people facing more social disadvantage also face worse access, quality, and experience of some types of NHS services’ (The Health Foundation 2022, 4). This was similarly echoed in the recent report by *The King's* *Fund* which found that healthcare services were not always reaching those in poverty (Mallorie 2024). The Lancet's detailed special issue also reported that ‘inequity in health‐care service provision is enduring and fundamental: an intractable concept that lies at the heart of the inverse care law’ (The Lancet 2021, 767).

It is important to understand how the ICL is impacting people who are marginalised. Evidence shows that those in the most deprived areas experience the greatest healthcare inequalities (NHS England 2023a), and this particularly impacts the north east of England (Office for National Statistics 2021). For instance, the population in the north east of England is 2.67 million people and over half a million people (581,276) are identified as living with fair to very bad health, with around 28.5% registered as disabled or living with long term physical or mental health conditions that impact their day to day activities (Office for National Statistics 2023b). Drug‐related deaths are also now at their highest recorded level nationally since records began (5448 deaths in 2023 and 11% higher than 2022), with the northeast continuing to have the highest number of drug‐related deaths for 11 years (174.3 deaths per million people) (Office for National Statistics 2024). Although people who use illicit drugs are not a homogenous group and can range across the socioeconomic scale, many in this population group do experience multiple disadvantage, complex co‐morbidities, and deep unmet needs. As such, we explore how people who use illicit drugs might be impacted by the ICL.

The ICL has consequences for social and health inequality, not least because inequalities have been estimated to cost £106.2 billion to the UK (The Equality Trust 2023). Total expenditure on healthcare in the UK in 2021 was £280.7 billion—which is around £4188 per person (Office for National Statistics 2023a). It is worth investing in solutions that address health inequity, not least to improve health outcomes but because this also has a broader economic benefit by reducing pressure and service demand on health and social care services. Ford‐Gilboe et al.'s longitudinal study (*n*‐395 participants, structured interviews, involving 4 primary health care clinics) examined the utility of equity orientated health care (EOHC) and found that EOHC is indicative of better patient health outcomes over time, with particular benefits for those in marginalised conditions (Ford‐Gilboe et al. 2018). As such, there is an economic and social justice benefit to understanding the persistence of the ICL and ingrained health inequity across health care (Darzi 2024).

The ICL helps us to shine a light on the harms arising out of the inequities that are embedded in healthcare systems (The Lancet 2021). Tudor Hart noted that middle class populations were often better served by health care services. They had greater expectations and higher demands of health care staff and were able to navigate complex health systems better in comparison to populations that are marginalised who, despite presenting with greater need and urgency, often experienced poorer quality care from staff, longer waiting times, fewer referrals, shorter consultation times, and incomplete explanations of diagnoses (Tudor Hart 1971). Tudor Hart described this simply as, ‘rich people get too much and the poor too little, and the same is true of medical care’ (Tudor Hart 1971, 411). These disparities in healthcare provision persist today and deepen health inequalities, perpetuating harm towards people at the sharp end of the inequalities spectrum, with severe consequences to ‘human flourishing’ (Addison, Lhussier, and Bambra 2023; Pemberton 2016).

Policymakers have attempted to address the ICL by injecting more financial capital into certain areas of high deprivation to maximise healthcare availability, targeting populations that are marginalised (The Lancet 2021; NHS England 2023b; The Health Foundation 2022). Currently, drug treatment and services vary between each region depending on what is commissioned by local authorities—this can be accessed via GPs and a referral to a specific drugs service, online support, apps, helplines, charities and private treatment services (NHS 2024). Investment in drug treatment services is also evident in the UK Drugs Strategy (HM Government 2021). There has also been a clear and concerted focus on operational strategy and integrated systems to reduce inequalities amongst groups who are marginalised by setting targets for improvement across continuity of care, vaccine uptake, health checks for severe mental illness, diagnosis and optimal management of key clinical areas. This is captured in *Core20PLUS5*, a national NHS England strategy which is intended to reduce healthcare inequalities through praxis and target setting at a national and system level (NHS England 2023b). This strategy focuses on the 20% most deprived areas and people who are marginalised within these locales (such as ethnic minorities, people with learning disabilities, long term health conditions, people with protected characteristics defined by the Equality Act 2010, and people who are socially excluded). These populations are further segmented in terms of people who fall into the category of ‘inclusion health’: ‘people experiencing homelessness, drug and alcohol dependence, vulnerable migrants, Gypsy, Roma and Traveller communities, sex workers, people in contact with the justice system, victims of modern slavery’ (NHS England 2023a). As such, we contend that *Core20Plus5* should be seen as a strategic attempt to address the ICL.

By making services more available and indeed accessible, this rationale attempts to address the impact of market forces on disadvantaged areas identified in the ICL. However, groups who are marginalised tend to engage less with healthcare services, experience poorer quality healthcare, have greater and more complex needs, and often have great difficulty navigating fragmented and complex systems (Dixon‐Woods et al. 2006; Mallorie 2024; Seddon and Ribeiro 2007). The complexity and increased fragmentation of the NHS and UK health and social care exacerbates challenges around patient data sharing, and ‘undermines a comprehensive and integrated care approach’, and in this regard inadvertently designs *in* systemic harms that disrupt continuity of care, impacting people who are already marginalised the most (Chan, Wright, and Majeed 2024). The resonance of the ICL today is further highlighted by the fact that individuals might not be accessing care for fear of being judged, blamed or shamed by healthcare staff—as a result, people from more disadvantaged backgrounds are accessing healthcare at a much later stage than other wealthier patients (The Health Foundation 2022; Mallorie 2024). Added to this, although health care services may be available, it is not always possible to access these due to financial constraints (e.g., transport, childcare and loss of earnings), meaning that those with the greatest need continue to be disadvantaged (Mallorie 2024). Those that do try to engage with healthcare services often do not get their needs fully met because of widespread pressure on the health sector, partly due to increased demand as well as more complex co‐morbidities in disadvantaged areas (Agenda Alliance 2023; The Health Foundation 2022).

COVID‐19 and the ‘c*ost of living crisis*’ in the UK have only exacerbated pressures on disadvantaged groups and healthcare services (Bambra, Lynch, and Smith 2021; Mallorie 2024), 6 in 10 people in the most deprived areas experience poor health and limited access to services, and mortality in areas with the lowest index of multiple deprivation are almost double that of the highest (Mallorie 2024). Building on this evidence base, our paper explores whether the *availability* of healthcare only partially explains and addresses the ICL. Little is yet understood about the role of the relational and structural dynamics of healthcare, how care is experienced and how stigma is tangled up with accessibility and healthcare inequalities.

Tudor Hart focused on availability of healthcare explicitly when outlining the ICL, but implicitly (and often overlooked) in his discussion was the recognition that quality of care, and interactions with healthcare staff, also impacted on what kind of experience patients received (Tudor Hart 1971). The quality of care determines whether a patient feels heard, understood and fully cognisant of next steps regarding treatment. Tudor Hart critiques Seale (1961) for labelling ‘time, convenience, freedom of choice, and privacy’, as the *frills* of healthcare that appeal to the middle classes, instead arguing that these are quintessential elements of quality care for all (Seale 1961, cited in Tudor Hart 1971, 40). The importance of relational aspects of care have since been studied in Roberts et al.'s work looking at how redesigning services to be more person‐centred for those living with long term conditions improves health outcomes and patient satisfaction (Roberts et al. 2019). However, health and social care services are still yet to prioritise these relational aspects of care and so the ICL means that disadvantaged areas miss out, and as Lord Darzi states: ‘There is much work to be done if quality of care is to become the organising principle of the NHS once more’ (Darzi 2024, 66). Yet, affluent populations can buy‐in or demand these so‐called ‘frills’ as part of their experience of *good quality* healthcare (Costa‐Font and Zigante 2016) but the same cannot be said of less advantaged patients. Middle class populations have also long understood the value of their combined social and knowledge capital and how to mobilise these in different spaces and around certain people (Bourdieu 1986, 1990; Bourdieu and Wacquant 2013) (e.g., across education settings and health care) to advance and meet their needs.

Tudor Hart writes that the willingness to explain, take more time during consultations and treat a person with courtesy can all be *guaranteed* if paid for, but he cautions us to be mindful that not being able to pay for or demand a quality experience does not signal an absence of desire amongst disadvantaged populations. UK patient satisfaction scores are used as an indicator of the quality of a patient's experience—*The Health Foundation* highlight that between 2015 and 2021 GP ‘practices serving the most deprived areas received the lowest overall patient satisfaction scores, while practices in the most affluent areas received the highest’ (2022, 12). In this regard, patients can experience inequities in health care via discrimination, stigma, discourtesy, and systems of healthcare which can be detrimental to their health and wellbeing and their likelihood to engage further with services.

We focus on stigma because it is embedded structurally and relationally into health services, impacting access to, engagement with, and experience of good quality healthcare. We conceptualise stigma as a *verb*—that is, harm that is done *to* individuals and communities by more powerful factions in society (Addison, McGovern, and McGovern 2022). Stigma constitutes many major and minor acts of symbolic violence that are weaponised by powerful groups and do harm to a person's health and wellbeing (Addison and Lhussier 2025; Addison, Lhussier, and Bambra 2023). Stigma operates at a micro and macro level and can come to be perceived, anticipated and/or internalised by an individual (Hatzenbuehler 2017; Addison and Lhussier 2025; Lochhead et al. 2024; McGovern, Addison, and McGovern 2024). Stigmatisation and associated harm constitute an ‘invisible reality’ that is often overlooked as a social determinant of health (Addison 2023; Addison, Lhussier, and Bambra 2023; Bourdieu 1990). Mechanisms of stigma often draw on intersections of identity that are considered of lower ‘social status’ by the prevailing dominant classificatory schema, so‐called deviant and risky practices, as well as place and space (Wacquant 2008). Stigmatisation is most frequently a demarcation of a power imbalance that tends to serve the interests and needs of a more dominant group by controlling and subjugating less powerful groups (Tyler 2020). Policymakers, professionals and the public can be unintentionally stigmatising and unaware of the damage this can do (e.g., compassion fatigue and microaggressions) (Wing Sue and Sanierman 2020). Likewise, systems and structures can be stigmatising because they do not function efficiently for certain groups, thereby marginalising them (Hatzenbuehler 2017), and consequently deepening unmet need.

In this paper we suggest that stigma is an important tenet of the inverse care law and helps to explain persistent inequities in healthcare and health inequalities for groups who are marginalised (i.e., people who use drugs), which cannot be explained by availability of services and market forces alone.

## 2 Research Design

This research study is qualitative in design and utilised semi‐structured, in‐depth interviews with *n*‐24 people (12 men, 11 women, 1 transgender); aged between 20 and 50 years old; majority White British sample due to the population demographics of the north east of England being 93.6% white (HM Government 2022). This research was located in the north east of England, which has a number of local authorities that fall into the lowest indices of multiple deprivation (Ministry of Housing Communities and Local Government 2019).

We used a purposive and snowballing sampling approach to include a range of voices and inclusion criteria requiring that at least one of the following class A or B illicit drug as a primary drug of choice (past or present use): heroin, crack or crack cocaine and spice (novel psychoactive substance), although a range of frequency of drug use, and differing levels of consumption were acceptable. We focused on this subpopulation because their experiences of care are often under‐heard and under‐served and have not been explored in relation to the ICL to our knowledge. All participants were over 18 years old, and the project was advertised via social media, leaflets, posters and third sector organisations who acted as gatekeepers and helped to establish contact with interested persons. Participants were advised that all discussions would be anonymised and identifiers removed and that taking part would be treated as confidential. All participants were offered a £10 shopping voucher.

Fieldwork occurred before and during the global COVID‐19 (C‐19) pandemic (2020–2021) meaning that a combination of face‐to‐face (*n*‐12, before C‐19) and online/telephone (*n*‐12, during C‐19) interviews were conducted to mitigate the risk of C‐19. The interviews did not generally focus on C‐19 in discussion.

Sound ethical practice was adhered to and approvals were given by the respective university ethics board. Participation in the study was voluntary and all interested persons were given the opportunity to look at an information leaflet about the study and ask questions.

Data was organised using NVivo and coded by MA (NVivo). Initial categories were established and then arranged into themes and discussed with the team. Our analysis was developed using Braun and Clarke's framework (Braun and Clarke 2006) drawing on a reflexive and iterative approach to test the credibility of our inferences.

## 3 Findings

In this section we draw on the experiences of people who use drugs and their interactions with healthcare staff and health systems to explore how stigma can be embedded structurally and relationally in health care provision, impacting their access to, engagement with, and experience of good quality healthcare. In doing so, we focus on the following themes arising out of the experiences of people who use drugs: (i) pressure on health and care services and long waiting lists, (ii) sensitivity to compassion fatigue from staff and impacts on engagement with support, (iii) complex systems that are difficult to navigate and (iv) stigma and drug use.

### 3.1 Pressure on Health and Care Services and Long Waiting Lists

Pressure, high demand and long waiting lists are all contributing factors to health harms and unmet need. This particularly impacts people whose health and care needs are generally more complex and who are faced with services that are over‐subscribed, under‐resourced and often focused on single issues. Participants in this study recognised that staff in drug treatment and healthcare services were experiencing high workloads.

I got a CPN [Community Psychiatric Nurse] pretty quick when I moved here, lovely lady, when she turns up, very busy, she is, she's really… they're all snowed under, they've got that many cases at the moment it's unreal.

(Kev, 41 year)

Fragmentation of health services into single issues for treatment can be problematic for patients with complex and intersecting needs that are not accommodated by the system (see Chan, Wright, and Majeed 2024). For instance, Chelsea illuminates the unintended stigma harms that can arise from very busy and pressured systems, and how this can impact on drug treatment, self‐esteem and recovery.

…as soon as they say I'm getting kept in, even before that I tell them I'm on Methadone, obviously, as soon as they say I'm getting kept in, I start, right, ‘Can you ring X to get my dose?’ do you know what I mean, ‘so you can give me it?’ ‘Yeah, no problem’. Then I ask them again, ‘Have you rang for the gear?’ ‘No, no, we'll do that’. ‘Have you rang the gear?’ ‘No, sorry, we'll do it’. ‘But it's closed now, can you ring the pharmacist?’ ‘All right, we'll do that’. Give all your information to the pharmacy. ‘Have you rang the pharmacy yet?’ ‘Oh no, we haven't done that, we'll do that’. And I know they're busy, but it happens every time I go into hospital. I never get my Methadone the first day, ever. So then I go out and get someone to bring my gear up to the hospital, and then I'm back to square one.

(Chelsea, 43 years)

Demand for drug treatment and healthcare has gone back to pre‐pandemic levels and in certain places, demand has increased because of the dual impact of COVID‐19 and the ‘cost of living’ crisis (Agenda Alliance 2023; Mallorie 2024). Some participants shared their anger and frustration with healthcare services because of the lack of continuity in their care. For instance, being assigned a keyworker, for that to then change multiple times, had a negative impact on Chelsea:

…you get used to one worker and then they fucking change you to another, which doesn't help you. It doesn't. My worker is still here, my old worker is still here, why can't I still see her? I don't understand. Oh, they swap you around because they want it to look better for their records, so they can… do you know what I mean? So they can get funding. Obviously funding is important, I know that. But I feel that you get passed about from pillar to post so it looks good on someone's worksheets.

(Chelsea, 43 years)

Chelsea became increasingly disengaged with her healthcare as a result and felt that the changes she experienced were a way for the service to manipulate performance data to access funding. Andy shared his weariness regarding time spent on NHS long waiting lists and treatment via medication. He discusses his experience of trying selective serotonin reuptake inhibitors (SSRI)—a type of antidepressant, but he felt this did not address his health needs.

Well, over the years I kind of tried it all really with the NHS and stuff. I've spent years on waiting lists and I haven't really got anywhere and then, because I've always self‐medicated I've tried a couple of SSRIs and things like that, they've just never really worked or they've made us feel a lot worse, and then I've just resorted back to self‐medicating, because that's worked.

(Andy, 29 years)

Some felt that staff wanted quick results that could be achieved by prescribing methadone. Haven shares how they wanted to address the causes of their drug use through talking therapies, which is more time intensive, but felt that the service was not designed in this person‐centred way. Treatment was granted as medication, and further support was conditional on a methadone prescription.

…when they heard, ‘heroin’, they were immediately, ‘Okay, do you want Methadone or Subutex?’ and I was like, ‘Well I don't really want to swap one drug for another, I've been doing that for ten years’. I want to resolve what's making me use drugs in the first place and that wasn't even on the table. To even get an appointment there I really had to be willing to go onto a script, and then, once I was on one there wasn't a lot else on offer and to be honest, they didn't seem to have high hopes for my treatment.

(Haven, 30 years)

The reasoning for this was not fully explained to Haven who was left feeling that their treatment options were highly limited and constrained by the healthcare system in place. Likewise, Samantha shares her frustration with staff and treatment services that perhaps at times hold unrealistic expectations of recovery for people who use drugs.

… they thought that I could just stop using like that, get on methadone, be on methadone for a few weeks and then come off it and be like normal and it can't be done like that, they didn't understand any of that.

(Samantha, 36 years)

Simply treating the physical addiction to heroin by prescribing methadone was not enough. Samantha gives a sense of the pressure and pace structured into these healthcare systems that are focused on targets and results. This pressure on workload, case turnover and performance culture had a dehumanising effect both on the staff who were overloaded with cases but also on the participants trying to access care.

Engaging with multiple services was also regarded as a factor which constrained patient autonomy and capacity to be involved in discussion and decision‐making around treatment pathways.

…bouncing around different services and trying to get help, it felt like decisions were being made about me rather than involving me.

(Haven, 30 years)

These factors are counter to Tudor Hart's argument whereby needs are met through higher quality interactions that include listening and ‘digging beneath the presenting symptom’ (1971, 40). Shorter appointments, longer waiting times, higher demand for treatment, medicated treatment pathways and lack of continuity all contribute to building in structural stigma towards already disadvantaged people.

### 3.2 Sensitivity to Compassion Fatigue

Some participants shared how their experiences of healthcare were negatively shaped by their interactions with staff who presented as fatigued and disengaged with their work. Although reasons for experiencing fatigue and burnout amongst staff can be attributed to increased pressures and demand, some of the participants felt that this reduced the compassion that staff were able to feel towards them, and their capacity to see them as human beings. This dehumanisation impacted their own self‐esteem and self‐belief that their treatment would work. Haven noticed that their healthcare provider did not have any belief that their efforts to titrate their methadone would be worthwhile, and instead the staff member undermined any potential gains by assuming that Haven would be topping up their dosage with illicit drugs.

I remember after I'd done my titration, I went for my first check‐up appointment with the clinician and at that point they're looking at if you're on the right dose, if it needs to be higher or lower, things like that, and the first thing they asked me when I got into the room was, ‘How much heroin are you using on top?’ There wasn't even a question of, ‘if’ I was using on top, you know? It was just the assumption that anyone who's put on Methadone is still using heroin and it just immediately put an idea in my head that these people don't really expect me to actually get any better, and actually at the time I'd been off heroin for about ten days I thought I was doing quite well and it just immediately made me think, ‘Well, why even bother trying?’

(Haven, 30 years)

Although it is important that healthcare staff are aware and mindful of patients ‘topping up’ during the titration process in case of over‐dosing, Haven perceived this particular risk to be poorly discussed with them, and they felt stigmatised in this interaction. The importance of sensitivity, compassion and *re*humanising within healthcare interactions should not be underestimated. For instance, Alan shares how low self‐esteem can be compounded by stigmatising interactions with fatigued staff who are dealing with high demands and pressure.

And when you're feeling that shit about yourself it's a bit like… if the one person that you're meant to be working with hasn't got that belief in you or gets sick of you, then what chance do you really have?

(Alan, 20 years)

Hannah also discusses her experience of healthcare interactions as transactional and lacking empathy. In this excerpt they draw attention to feeling unseen, unheard, and misunderstood, particularly in relation to the impact of trauma on their life:

I don't know if it's just not understanding maybe of what they've got themselves into and not empathetic, maybe not people persons, not understanding of trauma and the rest of it to be honest, and maybe not understanding the bigger picture that this medication isn't going to save everybody and that's going to be it, and it's a lot of work rebuilding people after being through stuff.

(Hannah, 35 years)

Treatment and care can be costly and time intensive, especially when addressing multiple, complex and unmet needs. In Hannah's discussion, she illuminates the speed in which interactions can happen and how this can generate unrealistic expectations of wellness and recovery. For her, there is little attempt to understand or heal the reasons that led to drug use in the first place—instead the focus is on treating the symptoms and not the cause, and this can feel transactional and ineffective.

### 3.3 Navigating Complex Systems

Stigma can be embedded in systems and structures by making it more difficult for certain people to navigate and access the help they need. The *Agenda Alliance* shows that women on probation missed appointments due to complexity in the system, misunderstandings and unclear instructions (Agenda Alliance 2023). Elsewhere, the King's Fund highlight the complexities of NHS systems and administration (e.g., travel expense claims, social prescribing) and how these issues disadvantage more deprived patients (The King's Fund 2024a). Haven shows how access can depend on where you live as well as other barriers:

Access to support services and resources is a postcode lottery for a lot of people and then there's further barriers put in the way if you don't fit the structure.

(Haven, 30 years)

Chang, Dubbin, and Shim (2016) describes cultural health capital (CHC) as a resource that helps people move around these complex systems, engage with healthcare professionals and get the treatments that they need. High CHC is beneficial because it constitutes a way of knowing how to ask for and get treatment. Hannah shares how she became familiar with the drug treatment system and knew how to use this knowledge (i.e., CHC) to her advantage to get access to the support she needed.

I knew the system and I knew how to get away with stuff […] obviously dual diagnosis, trying to get a dual diagnosis ‐ so I had to get well a bit and lie a little bit to get the medication I needed, and then once I got on that medication we started to have a bit lift off then, things started improving quite a bit.

(Hannah, 35 years)

Hannah goes on to say that she had to be persistent to access this support:

I'm not vocal but I am vocal, I sort of… I went on a mission and just banged on every door and got quite a few doors open to me because I wouldn't give up […] but then I still struggle at times.

(Hannah, 35 years)

Nevertheless, these efforts require emotional labour to access healthcare and navigate systems, which is illustrative of how systems and structures can be stigmatising, obstructive and harmful. Hannah goes on to say that she was able to rely on the support of her mother to help her overcome barriers and share the cognitive load whilst also being in poor health.

I was quite lucky to have people push to get me things where not many people's got that strength. Like I say, I was quite lucky to have my mum and people to fight my corner to make sure I got certain things whereas if I didn't have that I'd still be out there and I'd still be using drugs even more so.

(Hannah, 35 years)

Hannah notes how this social capital helped to fortify her efforts to keep persisting until she accessed the support she needed. Not everybody has relationships that they can draw on to navigate these systems. Alan recognises that, although treatment and healthcare may be available, it is not readily *accessible* to all:

I feel like I'd be able to access the same care that someone else would, I feel like the only difference would be that maybe if someone was lower class, their mum and dad maybe wouldn't be able to get them to a doctor's appointment as easy as mine would. Maybe I'd be advantaged in that way….

(Alan, 20 years)

### 3.4 Stigma and Drug Use

Stigma interactions can have negative impacts on a patients' engagement with a healthcare service, as well as their longer‐term treatment outcomes. Participants experienced relational stigma in interactions with staff, which affected the quality of their experience of care. Kev shares how he was sensitised to negative and stigmatising interactions with healthcare staff because of his drug use, and how this impacted his ability to get treatment for leg sores:

…the way you're treated, I mean for a lot of years I couldn't get proper treatment for my legs because they just look at you, ‘You're just a heroin user but that's your own fault’.

(Kev, 41 year)

Haven describes feeling criminalised by healthcare staff based on stereotypes of people who use drugs. Haven felt treated with suspicion when trying to make decisions about their treatment options and prescription delivery.

…it does feel like they're making an assumption about the life I lead if you've used drugs and for a while I was prescribed a restricted medication as well and I was basically treated like a criminal when I used to phone up for my repeat prescriptions, they were very suspicious if I asked for it to be posted out to me, because I was working full‐time.

(Haven, 30 years)

Haven goes on to explain in more detail how they experienced poor quality interactions with health care services because they were trying to seek a replacement prescription. Their stigmatised identity as a person who used drugs meant that staff automatically subscribed to negative stereotypes and assumed Haven was lying, and as such regarded to be a higher risk.

There was one time that my prescription got lost in the post and I had to speak to three or four different people to get a replacement one approved. They see me as a risk around medication and there seems to be the assumption still that I'm dishonest, which is very frustrating because I was always honest with them even in active addiction. I've never lied to a doctor about my drug use, I was always pretty up‐front and again, it's that stereotype. I guess maybe that's the stigma, maybe that is one area where stigma is playing a role. But again, it's the power behind it. I wouldn't care if all the doctors thought I was a liar and that I was full of diseases if they didn't have the power to control my access to care.

(Haven, 30 years)

The implications of this stigmatisation can have real consequences for people's ability to access care. These individuals can experience far greater restrictions around treatment options, as well as degrading interactions because of their status as a person who uses drugs that are not equivalent to the general population. These factors can be detrimental to health and recovery and can exacerbate unmet need.

It can take a great deal of courage to make a first disclosure of drug use and attempt to seek help. This moment can mean a person who uses drugs is very vulnerable and the response from healthcare professionals leaves a lasting impact on how a person engages in treatment going forward. Unfortunately, Karla shares a stigmatising interaction with her GP which meant that she was not only blocked from accessing treatment but she was also asked to leave her family GP surgery altogether:

By this point, I had a massive habit and that's when I started injecting and I went to the doctor's to come off it. Anyway, the doctor's who I went to, it was the first time really I admitted that I had a problem, so I went to see this doctor …and I was told to leave to find a new surgery, a new doctor's. I just think all my family's in that doctor's….

(Karla, 49 years)

This stigmatising interaction had a profound and long‐lasting effect on Karla. This is also echoed in Haven's account where they discuss the cumulative effect of ‘bad’ experiences with healthcare services which had a deleterious impact on their trust in staff and belief that treatment could work. These poor‐quality interactions can have serious implications for health and treatment outcomes, as Haven shows:

…the services that are there to help you do something about it, you might have been burned in the past. I had so many bad experiences in mental health services, the idea of trusting professionals to fix what was going on with me felt quite alien and when I did try, obviously I didn't get very far with them….

(Haven, 30 years)

Haven goes on to say:

Stigma can be annoying but stigma on its own doesn't have a lot of power, it's when you put the power of gatekeeping behind it and say, ‘People with this stigma attached to them can't have this’. That's when it becomes a problem.

(Haven, 30 years)

Although for some participants accessing support was considered challenging for various reasons, Chelsea did not feel access was the main issue;rather, she discussed interactions with her doctor to be particularly confrontational and stigmatising:

It's not accessing the support as such, it's the support you get. It's easy enough to access the support, but the doctor here – and this is not just my opinion, like – he's an arsehole. […] Doesn't like heroin addicts. I don't know why he's in this job. He's got an attitude with everyone who goes to see him.

(Chelsea, 43 years)

It can be difficult for some people to navigate healthcare systems and structures to access treatment, but for others, the service received can often feel stigmatising and of poor quality. Chang, Dubbin, and Shim (2016) show in their study of provider–patient interactions between people who use drugs and healthcare staff that participants tended to hold low CHC and received poorer quality care. Quality of interactions tended to increase if the patient was able to mobilise CHC to present a constructed performance of an ‘ideal’ and ‘deserving’ patient. In the following excerpt, interactions between Chelsea and her doctor deteriorated rapidly and her frustration is palpable. Chelsea is unable to mobilise enough CHC at this point to strengthen her position in her interactions with her doctor. Despite evidence and advocacy from her support worker Chelsea does not feel heard and is unable to get her needs met.

Then again, that dickhead doctor said because my sample was positive for opiates, he's putting me down. But my worker has taken a photo of my prescription and put it in my file so he could see what medication the doctors have given me, but he's saying that I shouldn't have took the codeine, knowing that they're going to show up positive in my sample. I said, ‘You can send it to the lab and see it's just codeine, not heroin’. ‘We're not going to pull the money out to do that’ he said. But you know I'm on fucking codeine from the doctor, the prescription's in front of you! Do you know what I mean? He just fucking winds me up, he really does. I hate him with a passion.

(Chelsea, 43 years)

These poor‐quality interactions mean that individuals may choose to disengage with treatment services that feel stigmatising and do not meet their treatment needs. This can have profoundly negative impacts on recovery as well as inadvertently widening health inequalities in this population. In contrast, the positive value of being seen, heard and included in decision‐making is clear in Hannah's account:

Empathetic, not patronising, just working with you and not against you, and I know we can be hard work and all of that, but just their caring nature and somebody to listen because half the time we're seen as hard work and I get that, but *we're not well* and our mental health will be suffering massively as a consequence of it. And I think just having someone to believe in you when you don't believe in yourself. I've always had people saying to me, even when I didn't think I'd make it, ‘You will make it, I believe you've got it in you’, and the more you keep hearing, the more people say that the more you start thinking, ‘Well maybe I can do this’, and not many people have got that.

(Hannah, 35 years)

Being treated with dignity and respect forms an important part of the experience of treatment and recovery, and helps to foster a more equitable health care service. High quality interactions shape a person's self‐belief and self‐efficacy and can have a positive impact on health outcomes. However, achieving high‐quality non‐stigmatising health care treatment and services is complex. Max draws attention to the ineffectiveness of healthcare systems that currently attend to the needs of populations who are marginalised:

A more person‐centred approach, you know? It's not a case of reinventing the wheel; it's about getting to the core and prioritising what people's needs and requirements are to the point of entry, whatever that basically may be within their adversity at the time. I know it's a big, big concept, but you know, I think we can all see that the systems are not working […] we're really trying to get to the core of how we can maybe make a better system for each and every one, you know? That's not just the basic kind of end user; that's the families, that's the professionals, it's all‐encompassing.

(Max, 45 years)

As Max discusses, a person‐centred approach that is non‐stigmatising is central to fostering high‐quality healthcare for marginalised people who use drugs. Addressing unmet need, ensuring that all people feel seen, heard and involved in decision‐making is essential to a good quality equitable healthcare system. Similarly, dealing with high treatment demand particularly amongst a population with multiple complex needs should be a key priority.

## 4 Discussion

We have discussed stigma across four themes to highlight the everyday harms that people who use drugs can experience in contact with health services. The structural and relational aspects of stigma embedded in healthcare reproduce inequities in *access to* and *experience of* good quality healthcare, which in turn impacts health inequalities. This is unfair and unjust.

The ICL states that ‘good medical care tends to vary inversely with the need for it in the population served’ (1971, 405). It helps to shine a light on the harms arising out of the inequities that are embedded in healthcare systems (The Lancet 2021). We know that populations living in marginalised conditions are chronically underserved by good quality healthcare and are unable to access health services which fully meet their urgent and complex needs (Lowther‐Payne et al. 2023; Matsuzaka, Romanelli, and Hudson 2021; Tudor Hart 1971). Although the ICL focuses mainly on availability of healthcare and the influence of market forces, we sought to broaden the discussion to include greater scrutiny of the inverse *experiences* of good quality health*care*. Our intention has been to expand what the *care* in ICL means, and how this is anchored in the relational aspects of care, whereas much of the research connected to the ICL so far has focused on the transactional aspects. Yet, the availability of good quality healthcare only partially explains the ICL. We sought to understand how the relational and structural dynamics of healthcare and systems can be stigmatising, negatively impacting a person's experience.

The relational and practical aspects of healthcare are very important to perceptions and experience of good quality healthcare (Liberati et al. 2022), but often these elements—such as longer appointments, shorter waiting lists, treatment options, autonomy, decision‐making, and having complex diagnoses explained fully—are denied to people who are marginalised because of stigma embedded in systems and structures. In our study, this meant that participants often felt they were not afforded the time and attention needed to fully meet and understand their complex needs, and this impacted their engagement with the service. The reasons for this more broadly can be traced to high demand for certain services (e.g., drug treatment, mental health) putting pressure on already stretched systems and staff, who then experience compassion fatigue and overwork. Furthermore, many of our participants demonstrated hypervigilance when interacting with staff and would often feel unseen and unheard if appointments were rushed. Some also reported being unsure about next steps regarding their treatment pathway. Others felt that their only treatment option was to take medication, despite expressing a desire to try more time intensive treatment such as talking therapies. This shows that quality of care can determine whether a patient feels heard, understood and fully cognisant of next steps regarding treatment.

We illustrated how stigma can be embedded in systems and structures that make it more difficult for certain people to navigate and access the help they need. Chang, Dubbin, and Shim's study (2016) of patient–provider interactions revealed how some patients were able to mobilise cultural health capital (CHC) to ensure entry and access to healthcare systems and that their healthcare needs were met. CHC also provides the ‘oil’ in social relations with staff, helping a patient to present as *deserving* of treatment. High CHC is beneficial because it constitutes a way of *knowing how* to ask for and get treatment, and knowledge of how complex systems operate. Some people in our own study were disadvantaged by CHC because they did not present themselves to healthcare staff in a way that conformed to the normative idea of a ‘good’ patient or ‘candidate’. This meant that some interactions were confrontational and detrimental for participants trying to access drug treatment. This stigmatisation, embedded in healthcare structures and services, can have dire consequences for health outcomes, meaning many individuals feel under‐heard and underserved and may disengage with services, exacerbating unmet health needs. This is critical to the ICL whereby good quality medical care varies inversely to need (Tudor Hart 1971). Metzl and Hansen offer a way to address stigma embedded in healthcare structures and services by encouraging reflective practice and improved clinical training for practitioners to strengthen their ‘structural competency’—that is, developing an understanding of the impacts of social and economic structures on patient health outcomes. They propose that by strengthening a practitioner's structural competency this means that interactions with patients become more compassionate and person‐centred rather than stigmatising (Metzl and Hansen 2014).

Our study shows that relational stigma affected patient‐provider interactions and impacted how participants would engage with services. Negative stereotyping and suspicion from staff meant that participants felt their treatment options were restricted, and their interactions felt degrading. Stigma is harmful to health and wellbeing because it is dehumanising and perpetuates an imbalance of power (Tyler 2020). It also impacts whether a person feels safe to disclose multiple co‐occurring issues that could be overlooked or missed if interactions with healthcare staff feel stigmatising or rushed. That said, participants in our study were able to identify good quality healthcare as an experience that helps to foster a non‐stigmatising interaction—this included empathy, compassion, and patience.

The main strength of this study lies in the inclusion of voices that have been marginalised and would otherwise be unseen and unheard—it gives prominence to what makes a ‘liveable life’ possible (Back 2007). A diversity of age and gender characteristics, drug use, and consumption patterns were sought; however, the main limitation of this sample is that it is largely white British; although this is congruent with the population composition in the northeast of England, the authors acknowledge that the themes discussed here are not necessarily reflective of experiences held by minoritised communities based on, for example, race. The authors were reflexive that the subject matter could be highly sensitive and emotionally activating for participants, so measures were taken to provide signposting to support, and the researcher endeavoured to be neutral and non‐judgemental throughout. The authors are mindful that whilst our discussion of inverse experiences of care relate to marginalised populations who use illicit drugs, further research is needed to understand overlaps and intersections within and across other marginalised groups.

## 5 Conclusion

Stigma is a key contributor to the inverse experience of good quality healthcare and requires greater attention from policymakers and healthcare professionals. Stigma impacts the quality of health*care* that marginalised populations have access to and receive, exacerbating unmet need which is harmful, unfair, and avoidable. We have discussed four key themes that illuminate the harms of stigma and add to the existing evidence underpinning Tudor Hart's Inverse Care Law, where *experiences* of good quality healthcare and services vary inversely to the need of the population served.

*The Health Foundation* state that addressing the ICL should be an explicit government priority. We build on this momentum and argue that *stigma* should be regarded as a key tenet of the ICL, forming part of the national health and social care strategy to reduce health inequalities amongst groups that are marginalised. The harms arising from stigma should not be underestimated so we would encourage health and social care services to prioritise and invest in basic training which raises awareness and understanding of inverse care experiences, and challenges stigma underpinning this. Furthermore, we encourage policymakers and healthcare professionals to apply care and support planning principles across all conditions and experiences, including mental as well as physical health, and addictions—this would begin to help address the inverse experiences of good quality healthcare for people who are marginalised.

## Author Contributions

**Michelle Addison:** conceptualization (lead), formal analysis (lead), funding acquisition (lead), methodology (lead), project administration (lead), resources (lead), writing–original draft (lead), writing–review & editing (equal). **Steph Scott:** writing–review & editing (equal). **Clare Bambra:** funding acquisition (supporting), project administration (supporting), supervision (supporting), writing–review & editing (equal). **Monique Lhussier:** writing–review & editing (equal).

## Ethics Statement

This research acquired ethical approval from Northumbria University at the time that the lead author was employed by this institution, 16.8.2019 (Submission Ref: 17304).

## Consent

All participants who took part in this study provided informed consent.

## Conflicts of Interest

The authors declare no conflicts of interest.

## Data Availability

Due to the sensitivity of this data regarding personal drug usage of participants, and given it is a small qualitative dataset, it is justifiable that the data are not made available.
